# A case report of delayed diagnosis of danon disease: Caused by a newly recognized mutation in the lysosome-associated membrane protein-2 gene

**DOI:** 10.1097/MD.0000000000022640

**Published:** 2020-10-02

**Authors:** Ying Zhang, Hang Ren, Shanshan Zhou

**Affiliations:** The Center of Cardiovascular Diseases, The First Hospital of Jilin University, Changchun, China.

**Keywords:** cardiomyopathy, danon disease, lysosome-associated membrane protein 2, sudden cardiac death

## Abstract

**Introduction:**

Danon disease is a rare X-linked dominant genetic disorder caused by defects in the lysosome-associated membrane protein 2 (LAMP2) gene. Unless treated, cardiogenic death is the main cause of mortality. This case report describes a 19-year-old man who was diagnosed with Danon disease and survived for 3 years from symptom onset to death. The mutation in his LAMP2 gene (p.Gly221Ilefs∗19) had not been previously reported.

**Patient concerns:**

A 19-year-old man patient was hospitalized for intermittent palpitations. He had no family history of cardiomyopathy, arrhythmia, or sudden cardiac death, but his sister had died of cirrhosis at age 12 years, but the exact cause of cirrhosis was unknown.

**Diagnosis:**

Exome sequencing and Sanger sequencing identified a novel missense mutation (p.Gly221Ilefs∗19) in the LAMP2 gene of the proband. This mutation was also detected in his mother, confirming the diagnosis of Danon disease.

**Interventions:**

The patient experienced various types of arrhythmia throughout the clinical process, including Wolff–Parkinson–White syndrome, non-sustained atrial tachycardia, atrial flutter, and third-degree atrioventricular block. He was therefore treated with cardiac ablation procedures and cardiac resynchronization therapy.

**Outcomes:**

The period from the onset of symptoms to the onset of heart failure was 2 years. The patient died of cardiogenic death during the third year, at age 22 years.

**Lessons:**

Danon disease is a rare disease that is difficult to recognize because of its hidden early manifestations. Early identification of its clinical symptoms can lead to early diagnosis and treatment.

## 1 Introduction

Danon disease is a metabolic disorder first described in a report on 2 boys with cardiomyopathy, skeletal myopathy, and intellectual disability.^[1]^ This rare X-linked dominant genetic disorder was found to be caused by defects in the lysosome-associated membrane protein 2 (LAMP2) gene. Because of its X-linked inheritance, males are affected both earlier and more severely than females.^[2]^ Many reports have substantiated the clinical triad of Danon disease, consisting of cardiomyopathy, myopathy, and intellectual disability. Additional clinical manifestations include retinal disease,[^3^,^4^,^5^] hepatic disease,[^6^,^7^] and pulmonary disease.^[8]^ Furthermore, studies in LAMP-2 knockout mice have shown that many organs are affected, including the kidneys, pancreas, small intestine, thymus, and spleen.^[6]^ At present, treatment interventions for Danon disease are mainly limited to the prevention of sudden death and heart failure through cardioverter defibrillator (ICD) implantation and heart transplantation. This report is of a case of a 19-year-old man with Danon disease who underwent cardiac ablation procedures and cardiac resynchronization therapy. The LAMP2 mutation in this patient had not been previously reported, making it a rare phenotype.

## 2 Case report

**Figure 1:**
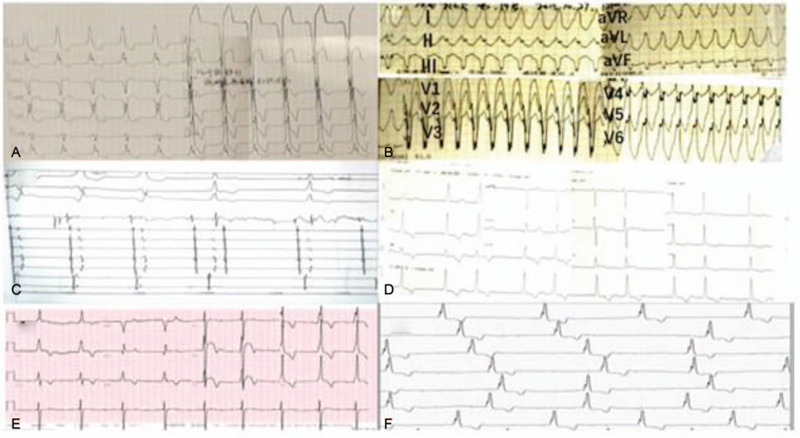
ECG. A, B: Surface ECG (paper sweep speed, 25 mm/s) at first admission, showing Wolff–Parkinson–White (WPW) syndrome with paroxysmal wide QRS tachycardia. C: Electrophysiological examination showed that the right side was bypassed, the AV was recently measured at 9–10 o’clock in the tricuspid annulus, and 30 W of energy was used to test the ablation at 60°C. The anterior passage of the bypass disappeared after 4 s, and the AV interval was prolonged in the intraluminal image. With II ° I AVB. D: ECG after radiofrequency ablation: II ° I AVB. E, 1f: Surface ECG (paper sweep speed, 25 mm/s) 2 years after initial radiofrequency ablation, suggesting atrial flutter, type B WPW, III ° AVB. ECG = electrocardiogram.

**Figure 2:**
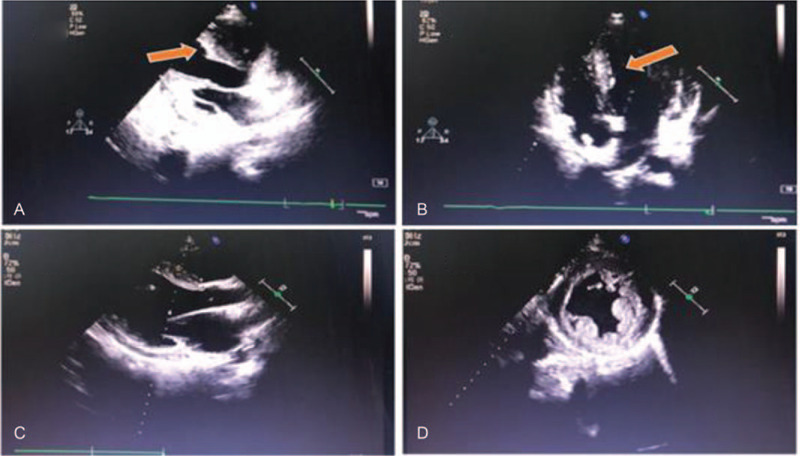
Echocardiography images. A, B: Thickening of the interventricular septum in the (A) long axis section of left ventricle and (2b) 4 chamber section. Initial septal thickness was 17 mm and initial thickness of the left ventricular wall was 13 mm. C, D: Echocardiogram showing that myocardial densification was not complete, that the left atrium and left ventricle were enlarged, the left ventricular beat was diffusely weakened, and systolic function was reduced. The left ventricular diastolic diameter was 68 mm, the left ventricular wall thickness was 9 mm, the septal thickness was 12 mm, and EF was 46%.

A 19-year-old man, previously in good health, was hospitalized for intermittent heart palpitations. There was no family history of cardiomyopathy, arrhythmia, or sudden cardiac death. His sister had died of cirrhosis at age 12 years, but the exact cause of this cirrhosis was unknown. A physical examination showed that this patient was hemodynamically stable. An electrocardiogram (ECG) showed Wolff–Parkinson–White (WPW) syndrome with paroxysmal wide QRS tachycardia (Fig. 1A and B). An echocardiogram revealed left ventricular wall thickening, suspected hypertrophic non-obstructive cardiomyopathy, and myocardial amyloidosis with an initial septal thickness of 17 mm and a left ventricular wall thickness of 13 mm (Fig. 2A and B). Pertinent laboratory test results (Table 1) included elevated serum concentrations of myo-inositol (667.5 ng/mL, normal < 107 ng/mL), cardiac troponin (CTnI; 0.187 ng/mL, normal < 0.05 ng/mL), creatine kinase-MB (5.40 ng/mL, normal < 4.3 ng/mL), aspartate aminotransferase (262.5 U/L, normal 15–40 U/L), and alanine aminotransferase (136.1 U/L, normal 9.0–50 U/L). During the process of radiofrequency ablation, the patient repeatedly showed atrioventricular block (AVB; Fig. 1C). However, after 3 radiofrequency ablation procedures, his WPW disappeared, but he continued to show second degree AVB (Fig. 1D).

**Table 1:**
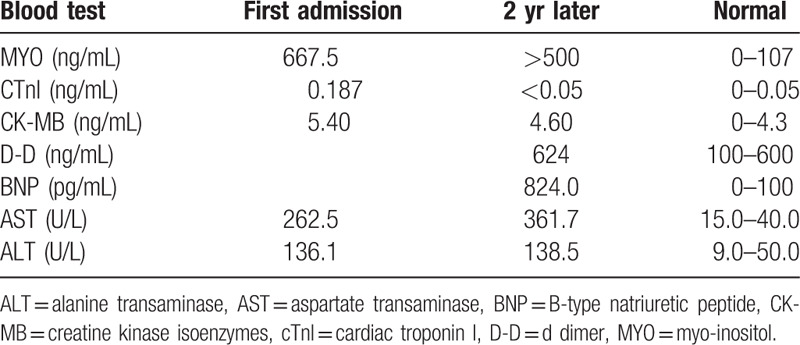
Blood test results.

**Figure 3:**
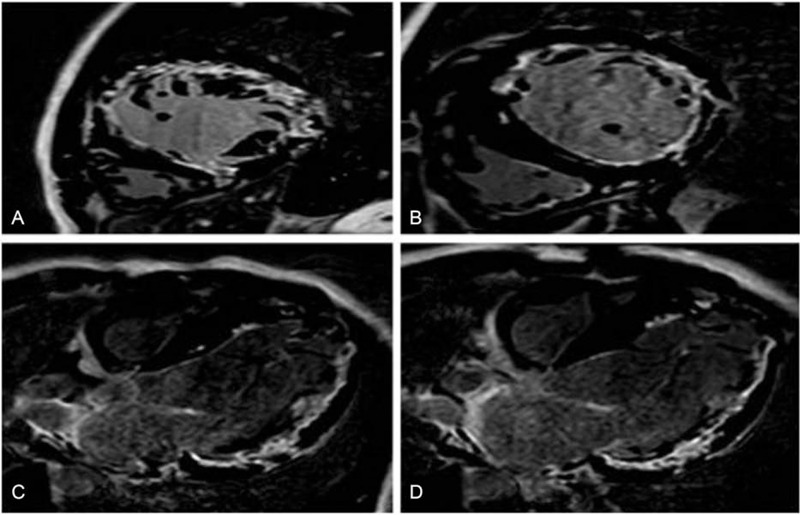
CMRI results. Results of myocardial perfusion, showing a diffuse subendocardial perfusion defect in the left ventricle and delayed enhancement of intracardiac contrast agent emptying. CMRI = cardiovascular magnetic resonance imaging.

**Figure 4:**
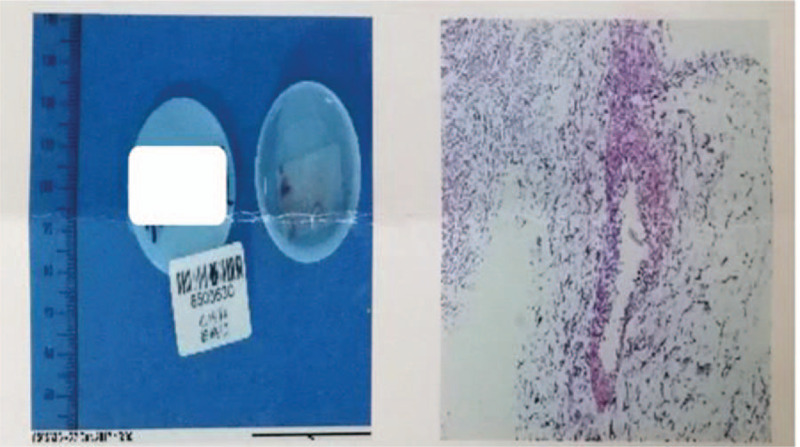
Congo red staining. Abdominal fat biopsy showing that the fat lobules were widened significantly and positively stained with Congo red (+).

Two years after radiofrequency ablation, the patient was again hospitalized for palpitations and dizziness. On physical examination, his apical beat was diffuse, his cardiac boundaries were enlarged to the left, and systolic murmurs were heard in the auscultation areas of his bicuspid and tricuspid valves. ECG indicated atrial flutter, WPW, and third-degree AVB (Fig. 1E and F). An echocardiogram showed that myocardial densification was incomplete, his left atrium and ventricle were enlarged, his left ventricular beat was diffusely weakened, and his systolic function was reduced. His mitral and tricuspid valves showed regurgitation and thickening of the interventricular septum, with a left ventricular diastolic diameter of 68 mm, a left ventricular wall thickness of 9 mm, a septal thickness of 12 mm, and an ejection fraction of 46% (Fig. 2C and D). Furthermore, cardiovascular magnetic resonance imaging (CMRI) indicated suspected amyloidosis of the cardiac muscle (Fig. 3). His laboratory test results had deteriorated since his previous admission, with his serum myo-inositol, creatine kinase-MB, aspartate aminotransferase, and alanine aminotransferase concentrations being higher than before, and his brain natriuretic peptide concentration being elevated (824.0 pg/mL, normal < 100 pg/mL) (Table 1). Amyloid cardiomyopathy was excluded by performing relevant laboratory tests and pathological diagnosis of the adipose tissue attached to the walls. Hyperplasia was observed in the fibrous tissue of the dermis, along with widening of the subcutaneous fat lobule space and positive Congo red staining (Fig. 4). These results did not support a diagnosis of amyloid cardiomyopathy (Table 2), and the transthyretin (TTR) gene was negative. A cardiac resynchronization therapy pacemaker was implanted to relieve the patient's clinical symptoms. Fifteen days later, he returned to our hospital because of a skin infection at the pacemaker implantation site and was given a permanent replacement pacemaker.

**Table 2:**
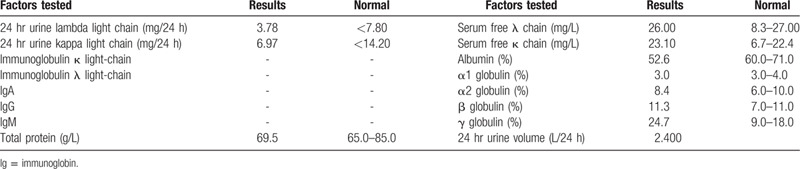
Laboratory tests for amyloidosis.

One month later, the patient was again hospitalized at our institution because of repeated aggravation of heart failure and the appearance of symptoms of restlessness and confusion. No abnormality was found on computed tomography of the head. No improvement was observed after symptomatic treatment, and he died of cardiac arrest.

**Figure 5:**
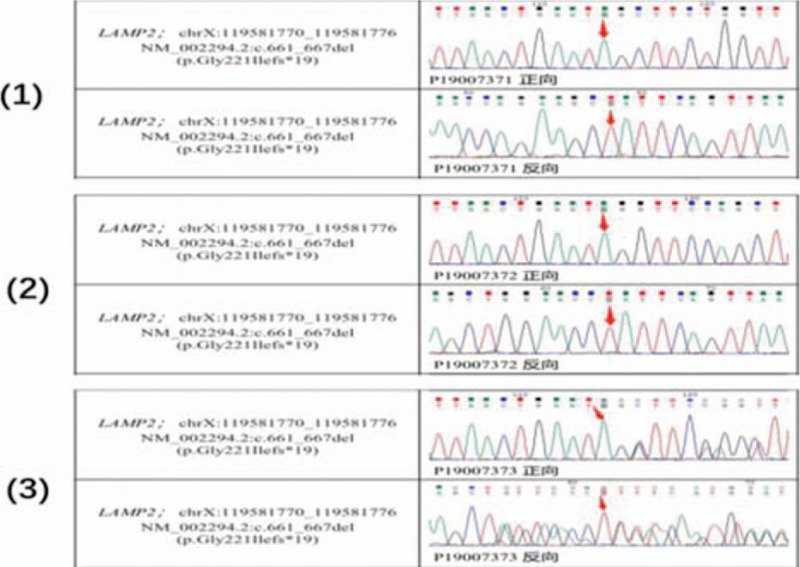
Sanger sequencing. Sanger sequencing showing that the proband (1) and his mother (3) carried the mutation in LAMP2, whereas the proband's father (2) did not. The position of the mutation in genomic DNA is indicated by arrows. The red arrows indicate the normal allele. DNA = deoxyribonucleic acid, LAMP2 = lysosome-associated membrane protein 2.

Subsequent genetic testing revealed that this patient was hemizygous for a previously unreported deletion mutation (p.Gly221Ilefs∗19) in the LAMP2 gene, confirming the diagnosis of Danon disease (Fig. 5). A myocardial biopsy was not performed.

## 3 Discussion

Danon disease is an X-linked dominant genetic disease. Its primary genetic defect, a mutation in the LAMP-2 gene, disrupts autophagy, leading to impaired fusion of lysosomes to autophagosomes, and the biogenesis of lysosomes.^[7]^ However, the exact function of the LAMP2 and the mechanisms by which LAMP2 deficiency causes Danon disease are not well understood.^[8]^ Because Danon disease has an X-linked mode of inheritance, clinical symptoms appear earlier and are more severe in male patients. Because men transmit this trait to all their daughters, more girls than boys should have Danon disease. However, because this disease is less severe in females, many go undiagnosed.^[7]^ The epidemiological characteristics of Danon disease are currently unknown, but it can affect any race or ethnic group.^[9]^ Although the clinical manifestations of Danon disease are a classic triad of cardiomyopathy, skeletal myopathy, and intellectual disability, its early clinical features are not obvious in most patients. Danon disease usually manifests initially as cardiac murmurs,^[10]^ chest pain,^[11]^ palpitations,^[12]^ and/or fatigue,^[13]^ all of which are easy to ignore. There are no currently established guidelines for the diagnosis or management of patients with Danon disease. Cardioverter implantation is necessary for patients with moderate to severe cardiomyopathy, symptomatic arrhythmia, and Danon disease with a family history of sudden early death. Radiofrequency ablation can provide temporary treatment for patients with arrhythmia, but it may not be effective because of diffuse myocardial fibers and the rapid progress of fibrosis. Heart transplantation should be considered early, especially in young men with rapidly progressing cardiomyopathy and a family history of sudden death.^[7]^ The prognosis for patients with Danon disease mainly depends on the severity of cardiomyopathy, with the need for cardiac transplantation being inevitable for most males in their second and third decades of life.^[2]^

The delayed diagnosis of this patient was due to our insufficient understanding of this rare disease. Apart from the classic triad, most patients with Danon disease are atypical and require careful screening. Arrhythmias are common in patients with Danon disease,^[7]^ affecting 86% to 100% of men.

Ventricular pre-excitation is the most common ECG abnormality in patients with Danon disease, with an incidence of 68.2% in men and 26.7% in women.^[14]^ Because of the presence of accessory pathways, the annulus fibrosus in most patients cannot completely separate the atria and ventricles, leading to ventricular pre-excitation. Danon disease associated with paroxysmal supraventricular tachycardia is also known as WPW syndrome.^[15]^ However, ventricular pre-excitation in some patients with Danon disease may be due to fasciculoventricular rather than atrioventricular pathways.^[16]^ The patient described in this report was admitted to the hospital with ventricular tachycardia. During radiofrequency ablation, AVB repeatedly appeared, indicating that the atrioventricular node was functioning poorly. Accelerated disease progression in this patient may have been caused by our cutoff of accessory pathways based on his symptoms. Furthermore, radiofrequency ablation in patients with Danon disease is not always successful due to diffuse and rapid fibrosis of the myocardium.^[7]^

Although ICDs are implanted toward the end-stage of the disease, sudden cardiac death may not be avoided. In 1 study of 7 patients who received an ICD, implantation ultimately failed to terminate fatal ventricular tachyarrhythmias in 5 patients.^[17]^ Therefore, early heart transplantation is necessary for patients with a high risk of sudden death.

In women, Danon disease usually manifests as isolated heart disease, whereas men may manifest damage to multiple systems.[^24^,^25^] Because the clinical manifestations of this disease are non-specific, an early diagnosis may be difficult. Our patient initially presented with cognitive and mental disorders, but a computed tomography examination of his head showed no organic disease. We also thought that this patient's mental symptoms were associated with the pain of this disease, without considering the mental abnormalities accompanying Danon disease. About 70% to 100% of affected men have learning disabilities and cognitive deficits, although they can learn to read, hold a job, build relationships, and usually live independently.^[9]^ In a study of 13 patients with Danon disease, nine had normal intelligence scores and only 1 had an intellectual disability. However, 69% of patients with this condition have been reported to meet the diagnostic criteria for at least 1 mental disorder, usually emotional and anxiety disorders.^[26]^ Other mental disorders can include measurement disorders, language disorders, attention deficits, and behavioral problems.^[9]^ Although studies have reported cognitive problems,[^14^,^27^] these problems have not been described in detail.

This patient experienced symptoms of fatigue during the later stages of disease. Abnormally elevated creatine kinase concentration and skin rupture at the site of the ICD suggest muscle damage. The liver may be a component of skeletal myopathy, leading to an increase in liver enzymes.^[5]^ About 80% to 100% of patients with Danon disease have skeletal muscle disease,[^25^,^28^] with the common clinical manifestations being asthenia^[29]^ and exercise intolerance.^[28]^ Severe skeletal muscle weakness and respiratory failure have also been observed in patients with Danon disease,^[30]^ with men having levels of creatine kinase 3 to 35 times higher than normal.

In addition, we neglected the results of relevant auxiliary examinations. Color Doppler echocardiography in our patient showed that the left ventricular wall was thickened. As Danon disease progresses, the left atrium and left ventricle become enlarged, gradually reducing systolic capacity. Cardiomyopathy has been reported as progressive in patients with Danon disease, with the typical manifestation being hypertrophic cardiomyopathy (HCM). Moreover, ejection fraction and lumen diameter often remain unchanged during the early stages of disease. After age 26 years, 11% to 12% of men with this condition develop dilated cardiomyopathy.^[9]^ In addition, the types of cardiomyopathy differ by sex, with most men having HCM, and similar percentages of women having HCM and dilated cardiomyopathy.[^14^,^18^,^19^,^20^] In a study of 197 patients with left ventricular hypertrophy of unknown cause, ten patients were confirmed to have electrocardiographic pre-excitation by next generation sequencing, with 3 having different mutations of the LAMP2 gene.^[15]^ These findings suggest that Danon disease should be highly suspected in patients with left ventricular hypertrophy and ventricular pre-excitation, 1 of the clinical manifestations we neglected.

Cardiac CMRI may have potential clinical utility and provide valuable morphological and structural information in patients with Danon disease. The late gadolinium enhancement (LGE) observed in Danon disease does not seem to be typical in patients with other causes of left ventricular hypertrophy. For example, LGE in patients with amyloidosis usually manifests as global transmural or subendocardial LGE with a characteristic “zebra” pattern. In patients with sarcoidosis, LGE has been found to involve the basal and/or mid-ventricular septum, whereas, in patients with Anderson-Fabry disease, LGE frequently involves the inferolateral segments with intramyocardial distribution.[^21^,^22^] CMRI found that all patients who had been diagnosed with Danon disease and the HCM phenotype had LGE that included the anterior, posterior, or lateral wall, with a subendocardial, intramyocardial, or transmural distribution. Only 1 patient (20%) showed involvement of the interventricular septum.^[21]^ CMRI in the present patient showed LGE involving the diffuse subendocardial and intermural walls of the left ventricular free wall and the muscular wall of the right ventricle wall.

Our patient was found to have rare ventricular septa, different from the involved sites of other patients with the HCM phenotypes. The range of involvement in our patient was uncommon pathologically. These findings indicate that the LAMP2 gene should be analyzed as soon as possible in patients with HCM phenotypes and in LGE patients with other pathologically abnormal distributions.

In addition to the increase in brain natriuretic peptide, cTnI was also increased in our patient. Increased cTnI has been observed during the acute stage of cardiac symptoms, especially in cases of rapid death. Recent reports indicate that an increase in cTnI has predictive value, although large-scale clinical studies are required to confirm this possibility.^[23]^

What has always confused us is the results of both color Doppler echocardiography and CMRI suggested the possibility of cardiac amyloidosis, as the latter is caused by abnormal protein (amyloid) deposition in cardiac tissue.^[31]^ The monoclonal immunoglobulin light-chain (AL; or primary systemic) type and (TTR; formerly senile) type are most common in cardiac amyloidosis.^[32]^ Because AL amyloidosis is mainly related to plasma cell disease, the number of relevant laboratory tests was increased, suggesting that the results in this patient were not consistent with AL amyloidosis. Moreover, this patient was negative on the TTR gene test, excluding the likelihood of TTR amyloidosis. When laboratory results are unclear, histological examination remains the diagnostic standard for amyloid cardiomyopathy. In addition to Congo red staining being positive, polarized light microscopy of amyloid fibrils stained with Congo red yielded characteristic apple green birefringence and intense yellow-green fluorescence with thioflavin.^[31]^ Unfortunately, we only performed Congo red staining, and the abdominal fat biopsy of this patient was positive. In addition to amyloid, other positively stained tissue elements include elastin, collagen, and eosinophils.^[33]^ However, the reason for the positive Congo red staining in this patient is uncertain.

Danon disease is a rare X-linked dominant disease, caused by mutations in the LAMP2 gene located at Xq24-q25.^[10]^ At least 110 different mutations in the LAMP2 gene have been detected, with the c.926gG>A mutation (causing exon 7 skipping) being the most common.^[7]^ The p.Gly221Ilefs∗19 mutation in the present patient had not been previously reported, suggesting it is a rare phenotype. Although this gene was from the patient's mother, and women are more likely to inherit Danon disease than men, the cause of his younger sister's death remains unclear. Because clinical symptoms are less severe in women with Danon disease, the disease is more insidious and cannot be diagnosed without gene diagnosis. Therefore, early molecular diagnosis and genetic counseling are very important for diagnosis and treatment.

The patient described in this study lived 2 years from the onset of symptoms to the onset of heart failure, and died of cardiogenic death in the third year. Patients with Danon disease often progress from being asymptomatic or having imperceptible symptoms to rapid deterioration and heart failure.^[15]^ Greater understanding of Danon disease may help with its early diagnosis and active treatment, especially if early heart transplantation can prolong survival.

## Author contributions

**Data curation:** Hang Ren.

**Software:** Hang Ren.

**Supervision:** Shanshan Zhou.

**Writing – original draft:** Ying Zhang.

**Writing – review & editing:** Ying Zhang, Shanshan Zhou.
